# Peritumoral endothelial indoleamine 2, 3-dioxygenase expression is an early independent marker of disease relapse in colorectal cancer and is influenced by DNA mismatch repair profile

**DOI:** 10.18632/oncotarget.25393

**Published:** 2018-05-18

**Authors:** Annabel Meireson, Inès Chevolet, Eva Hulstaert, Liesbeth Ferdinande, Piet Ost, Karen Geboes, Marc De Man, Dirk Van de Putte, Laurine Verset, Vibeke Kruse, Pieter Demetter, Lieve Brochez

**Affiliations:** ^1^ Department of Dermatology, Ghent University Hospital, Ghent, Belgium; ^2^ Department of Pathology, Ghent University Hospital, Ghent, Belgium; ^3^ Department of Radiation-Oncology and Experimental Cancer Research, Ghent University Hospital, Ghent, Belgium; ^4^ Department of Gastroenterology and Digestive Oncology, Ghent University Hospital, Ghent, Belgium; ^5^ Department of Gastrointestinal Surgery, Ghent University Hospital, Ghent, Belgium; ^6^ Department of Pathology, Erasme Hospital, ULB, Brussels, Belgium; ^7^ Department of Medical Oncology, Ghent University Hospital, Ghent, Belgium; ^8^ Immuno-Oncology Network Ghent (ION Ghent), Ghent, Belgium; ^9^ Cancer Research Institute Ghent (CRIG), Ghent, Belgium

**Keywords:** indoleamine 2,3-dioxygenase, colorectal cancer, immune checkpoints, microsatellite instability, MSI

## Abstract

Targeting immune checkpoint molecules has become a major new strategy in the treatment of several cancers. Indoleamine 2,3-dioxygenase (IDO)-inhibitors are a potential next-generation immunotherapy, currently investigated in multiple phase I-III trials. IDO is an intracellular immunosuppressive enzyme and its expression/activity has been associated with worse prognosis in several cancers.

The aim of this study was to investigate the expression pattern of IDO in colorectal cancer (CRC). In a cohort of 94 CRC patients, primary tumors (PTs) with corresponding tumor-draining lymph nodes (TDLNs, *n* = 93) and extranodal/distant metastases (*n* = 27) were retrospectively analyzed by immunohistochemical staining for IDO, CD8 and Foxp3. 45 MSS and 37 MSI-H tumors were selected to compare IDO expression, as these tumors are considered to have different immunogenicity.

A highly consistent expression pattern of IDO was observed in the PT, TDLNs and metastases, indicating that immune resistance may be determined very early in the disease course. IDO was expressed both by tumoral cells and host endothelial cells and these expressions were highly correlated (*p* < 0.001). IDO expression was observed more frequently in the MSI-H subset compared with the MSS subset (43% vs 22% for tumoral expression (*p* = 0.042) and 38% vs 16% for endothelial expression (*p* = 0.021)). Endothelial IDO expression was demonstrated to be a negative prognostic marker for recurrence free survival independent of disease stage and DNA mismatch repair (MMR) status (HR 20.67, 95% CI: 3.05–139.94; *p* = 0.002). These findings indicate that endothelial IDO expression in primary CRC, in addition to the MMR profile, may be helpful in disease stratification.

## INTRODUCTION

Colorectal cancer (CRC) is one of the leading causes of cancer-related deaths worldwide [1]. Current treatment regimens rely on combinations of surgery and chemotherapy, radiation therapy and/or targeted therapy (anti-EGFR and anti-angiogenic therapies), depending on TNM staging (American Join Committee on Cancer, AJCC) [2]. 5-year survival rates vary from 85–100% in local disease (stage I & II) to 30–40% in regional disease (stage III) to less than 5% in systemic disease (stage IV) [3].

Insights in the complex immune regulation at the level of the tumor microenvironment with subsequent development of immune checkpoint inhibitors have revolutionized treatment of several cancers. Durable responses and improved survival rates with programmed cell death-1 (PD-1)-inhibitors have been observed in malignant melanoma, non-small-cell lung cancer (NSCLC), renal cell carcinoma and bladder cancer [4–6], changing the therapeutic field in these cancer types significantly. Numerous clinical trials are currently investigating these agents in other cancers, including CRC. Despite these encouraging results, the objective response rate (ORR) in patients under immunotherapy remains incomplete and durable responses are attained in a limited subgroup.

Indoleamine 2, 3-dioxygenase (IDO) may be a new target amenable to inhibition in the treatment of several cancers. IDO is an immunosuppressive intracellular enzyme that initiates the catabolism of the essential amino acid tryptophan to kynurenine and its downstream metabolites. IDO has been demonstrated to be an endogenous mechanism of acquired peripheral immune tolerance *in vivo*, which can act as a mechanism facilitating immune escape in cancer [7]. Expression of IDO has been associated with worse prognosis in several tumor types, amongst which melanoma. A highly consistent expression pattern of IDO was observed in the primary tumor, the sentinel lymph node and metastatic tissues of melanoma patients, indicating that immune suppression is determined very early in the disease course of these patients [8].

IDO may be a general principle of acquired immune tolerance, implicating that IDO checkpoint inhibition might prove beneficial in various cancer types. Several phase I/II clinical trials with different IDO-inhibitors are under way [7]. Epacadostat (INCB024360, Incyte) and indoximod (NLG-8189, NewLinkGenetics), two orally available IDO-inhibitors, have both been demonstrated in a phase I study to be well tolerated in patients with advanced malignancies [9, 10]. In colorectal cancer, four phase I/II studies with epacadostat combined with PD-1 inhibitors are currently recruiting patients.

Several independent studies revealed a negative prognostic value of IDO in CRC. IDO expression in the PT has been reported to correlate with development of lymph node metastases and shorter overall survival [11]. A negative impact on survival was also observed with IDO expression in tumor draining lymph nodes (TDLNs) [12]. Moreover, strong IDO expression at the primary seemed to be associated with development of metachronous metastases [13–15]. Paradoxically, tumoral IDO expression was recently reported to be a positive prognostic marker on disease-free survival in microsatellite instable (MSI-H) CRCs [16].

The aims of this study were to investigate (1) the consistency of IDO expression in the PT, TDLN and metastatic tissue, (2) a correlation of IDO expression with tumor-infiltrating lymphocytes (TILs) at the PT, (3) differences of IDO expression according to the DNA mismatch repair (MMR) profile, (4) the prognostic influence of IDO on recurrence free survival (RFS).

## RESULTS

### IDO expression is highly consistent across disease stage

**Figure 1:**
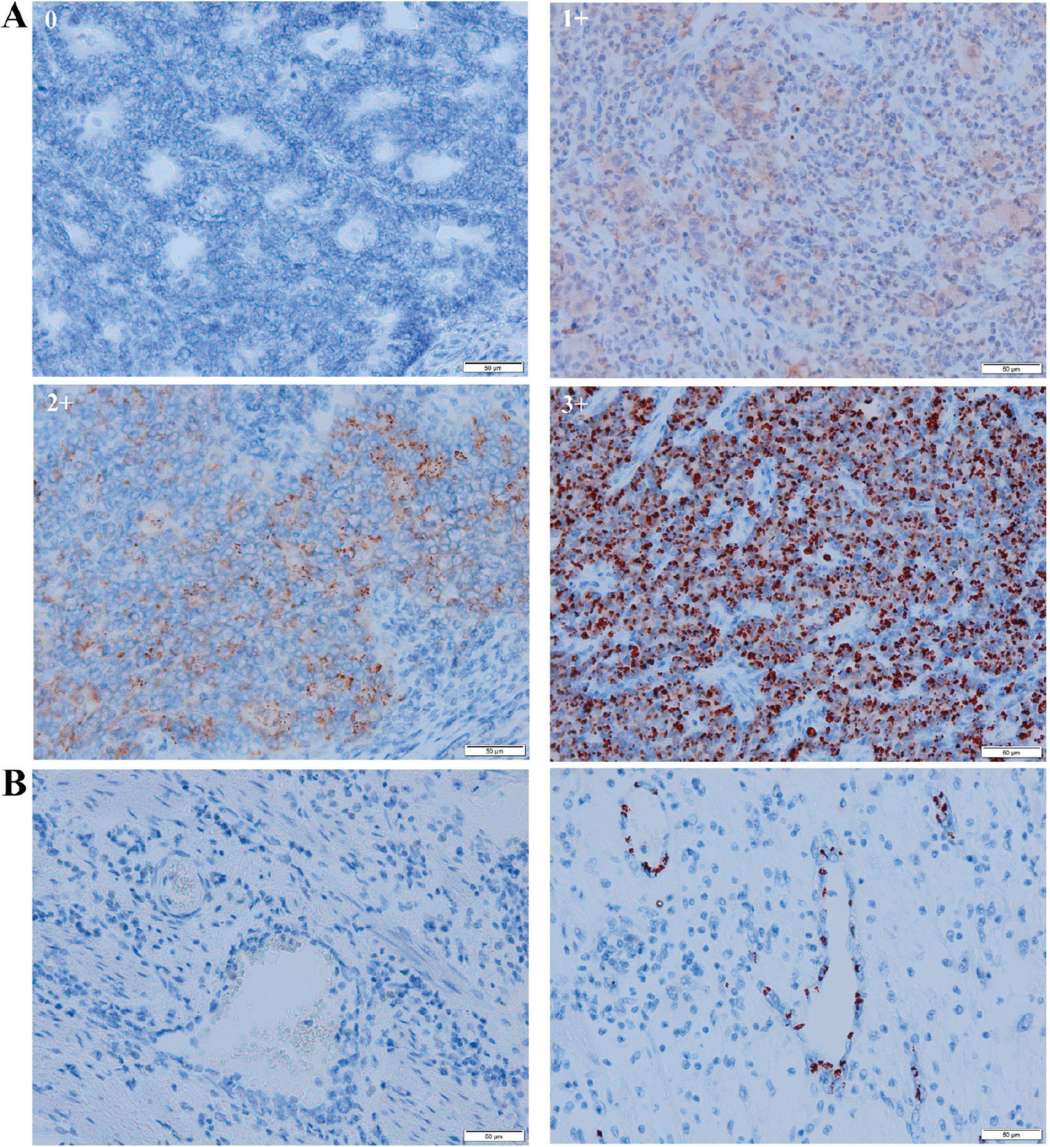
Representative scoring system of tumoral and peritumoral endothelial IDO immunohistochemistry in the primary tumor (**A**) no expression (0), weak expression (1+), moderate expression (2+) and strong expression (3+). For further analysis, tumoral IDO expression was dichotomized into an IDO-low expressing group (0 and 1+) and an IDO-high expressing group (2+ and 3+). (**B**) absence (left) and presence (right) of IDO expression by endothelial cells in the primary tumor.

In the PT, two major patterns of IDO expression were identified. A paranuclear dot-like IDO staining could be observed in neoplastic cells (Figure 1A). High IDO expression in tumor cells (2+ or 3+) was detectable in 30.9% (29/94) of cases. IDO could also be observed in endothelial cells in the peri-tumoral stroma in 25.5% (24/94) of patients (Figure 1B). Complete concordance of tumoral and endothelial IDO expression was seen in 84.0% (79/94) of patients, illustrating an association between these 2 distinct expression patterns in the PT (*p* < 0.001). Tumoral IDO expression did not significantly vary across disease stage at diagnosis: stage I: 27.3% (3/11), stage II: 27.6% (8/29), stage III: 31.4% (11/35) and stage IV: 36.8% (7/19) (*p* = 0.901). Neither did IDO expression by endothelial cells: stage I: 45.5% (5/11), stage II: 13.8% (4/29), stage III: 28.6% (10/35) and stage IV: 26.3% (5/19) (*p* = 0.204). In the TDLNs, a similar tumoral and endothelial IDO expression pattern was observed. There was a strong association between tumoral and endothelial IDO staining in the TDLNs (*p ≤* 0.001). Endothelial IDO positivity was present in 25.6% (11/43) of the TDLNs that were tumor-free. In 10 patients for whom both tumor-uninvaded and invaded TDLNs were available, a significant association in endothelial IDO expression was seen (*p* = 0.022).

For analysis of IDO consistency, TDLNs of 93 cases and distant metastases of 27 cases were available. Both endothelial and tumoral IDO expression in the PT were consistently expressed in the TDLNs (resp. *p* < 0.001 and *p* < 0.001) and metastatic tissue (resp. *p* = 0.009 and *p* < 0.001).

### IDO expression and the local immune infiltrate

**Figure 2:**
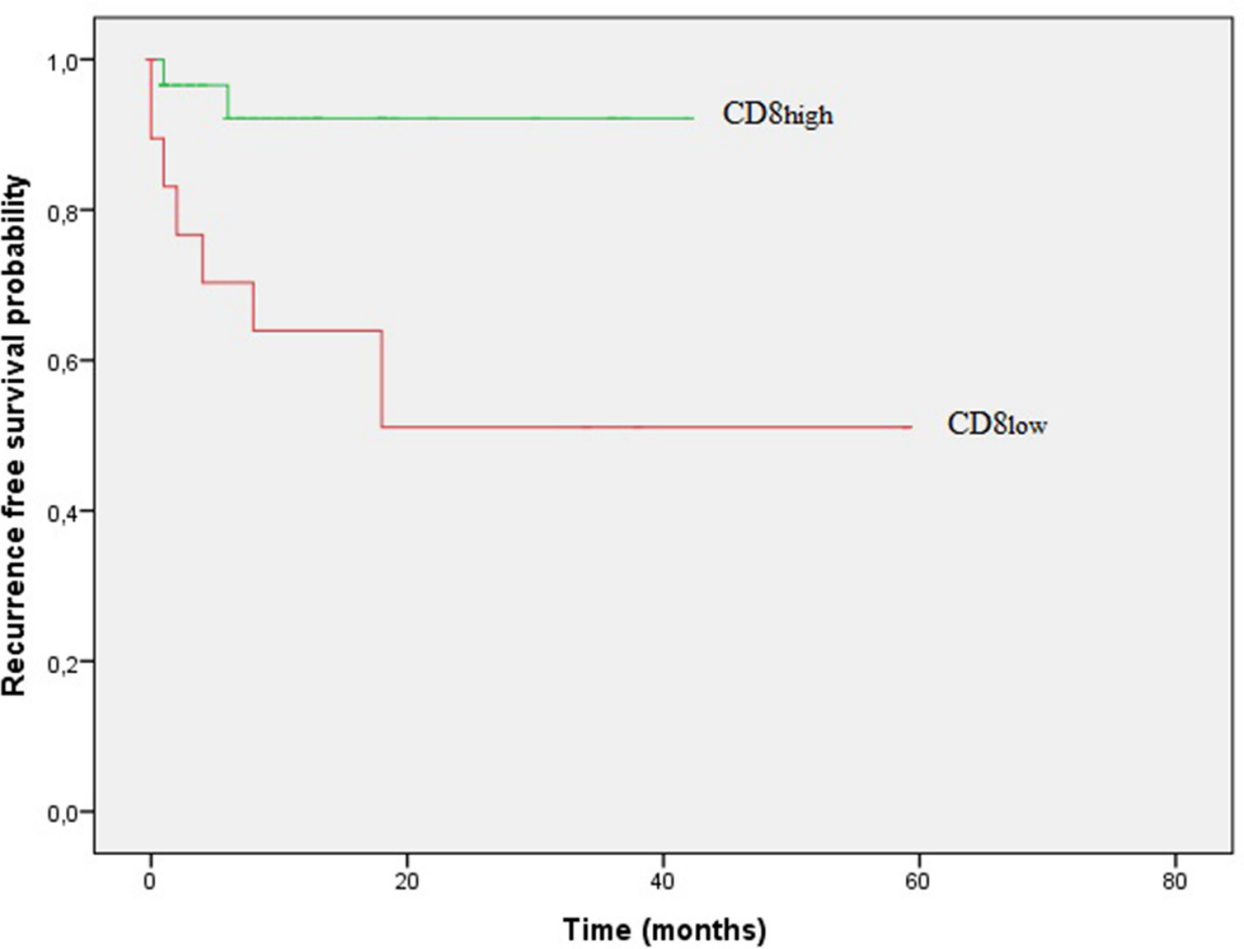
Kaplan–Meier curves of recurrence free survival (RFS) according to frequency of CD8+cells in stage I–III patients

CD8+ cells were more frequently present in MSI-H tumors (*p* = 0.010). ROC analysis was performed to dichotomize CD8+ cells in a ‘low’ and ‘high’ subset (cut-off value 87.96 cells/mm^2^). Using this cut-off value, a highly significant prognostic role for CD8 on RFS was observed (Figure 2, log-rank test, *p* = 0.004, cohort 2).

No significant differences in tumoral CD8+ cells could be observed in tumors with ‘high’ versus ‘low’ tumoral IDO expression in the PT (*p* = 0.963) nor in tumors with present versus absent endothelial IDO expression (*p* = 0.577).

No associations of Foxp3 with MMR status, CD8 cell count or tumoral/endothelial IDO expression were observed.

### IDO expression is correlated with MSI status

Microsatellite instability was highly correlated with proximal tumor location (*p* < 0.001).

Moderate or strong tumoral IDO expression was present in 43.2% (16/37) of MSI-H tumors compared to 22.2% (10/45) of MSS tumors (*p* = 0.042). Endothelial IDO expression in MSI-H tumors was also significantly higher compared to MSS (37.8% (14/37) versus 15.6% (7/45), *p* = 0.021).

### Prognostic impact of IDO expression

The prognostic relevance of IDO expression for RFS was evaluated in stage I-III patients of cohort 2. Tumoral IDO expression did not affect RFS (*p* = 0.548, log-rank test). Patients with endothelial IDO expression had shorter RFS time compared to patients without endothelial IDO expression (mean RFS: 41 months (95% CI: 26.19–55.64) compared to mean RFS: 61 months (95%CI: 51.71–70.88); *p* = 0.143, log-rank test).

Multivariate analysis with a Cox proportional hazard regression model including TNM stage, endothelial IDO expression and MMR status demonstrated that endothelial IDO expression (HR: 20.67, 95% CI: 3.05–139.94; *p* = 0.002) and MSS status (HR: 24.62, 95% CI: 2.38–254.36; *p* = 0.007) both negatively affect RFS, independent of each other and of disease stage (Table 1, upper panel). Moreover, endothelial IDO expression predicted relapse independent of the CD8 count (Table 1, lower panel).

**Table 1:** Multivariate Cox regression model for recurrence free survival

				95% Confidence Interval for HR		
Recurrence free survival		Coefficient	HR	Lower	Upper	*P*-value
**Clinical stage at diagnosis**						
	Stage IV	(reference)	-	-	-	0.037
	Stage I	−19,063	0.000	-	-	0.973
	Stage II	−3,360	0.035	0.002	0.512	0.014
	Stage III	−4,254	0.014	0.001	0.251	0.004
**Endothelial IDO expression**						
	Absent	(reference)	-	-	-	-
	Present	−3.029	20,670	3,053	1,39,943	0.002
**MMR status**						
	MSI-H	(reference)	-	-	-	-
	MSS	3,204	24,626	2,384	2,54,358	0.007
**Endothelial IDO expression**						
	Absent	(reference)	-	-	-	-
	Present	−1.578	0.206	0.054	0.789	0.021
**CD8**^+^**cells/mm^2^**						
	Low	(reference)	-	-	-	-
	High	1,930	6,891	1,422	33,399	0.017

Abbreviations: HR, hazard ratio; IDO, indoleamine 2,3-dioxygenase; MMR status, DNA mismatch repair status; MSI-H, microsatellite unstable; MSS, microsatellite stable.

**Figure 3:**
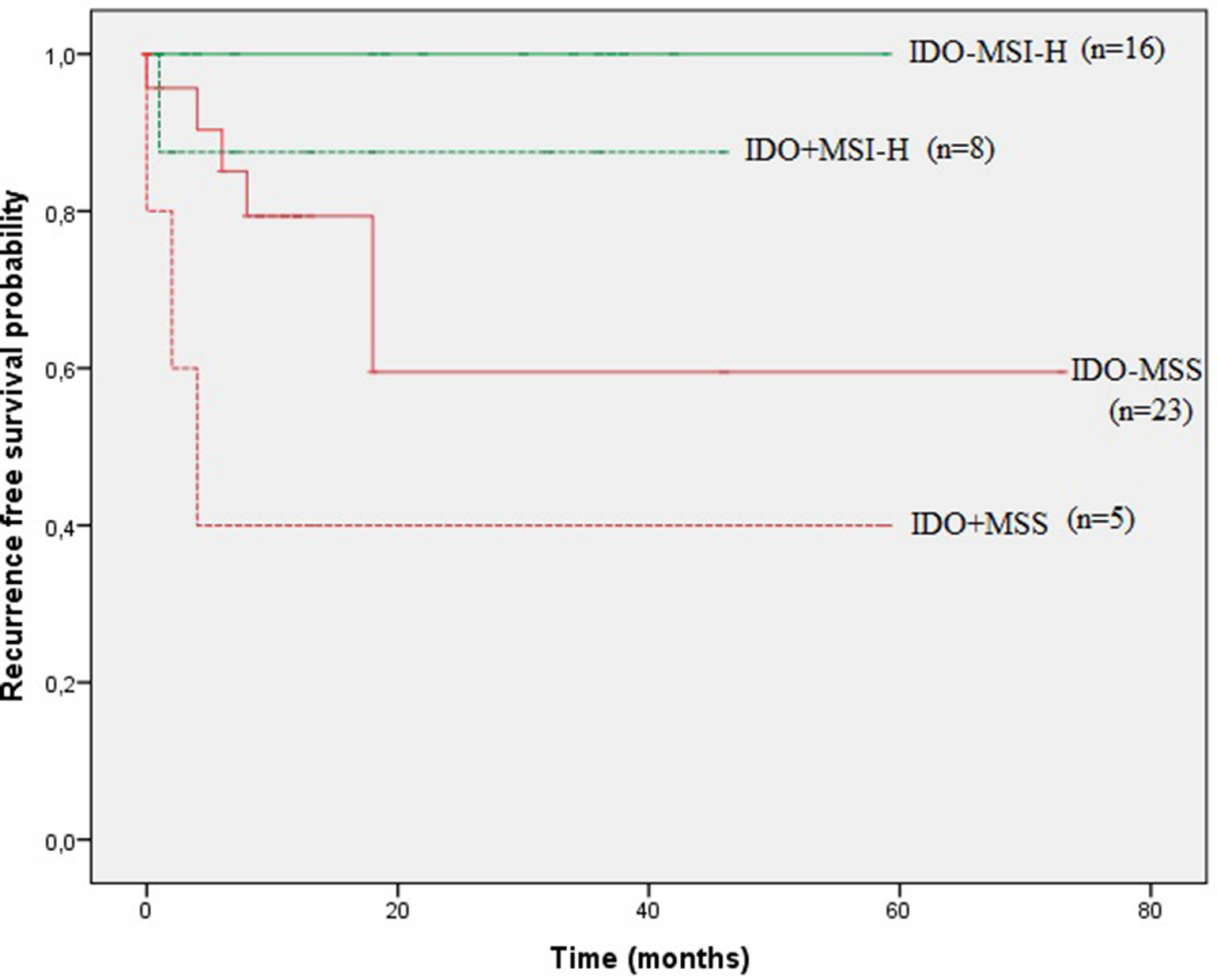
Kaplan–Meier curves of recurrence free survival (RFS) according to endothelial IDO expression and MMR profile in stage I-III patients

In order to estimate the combined prognostic impact of endothelial IDO expression and MMR status, patients were subdivided in 4 groups: IDO negative MSS/IDO positive MSS/ IDO negative MSI-H or IDO positive MSI-H. Univariate log-rank survival analysis demonstrated divergent RFS curves for the 4 subgroups subgroups (Figure 3: stage I-III patients, *p* = 0.010). IDO negative MSI-H showed the most favourable prognosis (3.5 year RFS of 100%), whereas IDO positive MSS had the worst outcome with a median RFS of 4 months (95% CI: 0.00–8.29).

## DISCUSSION

Study of the tumor microenvironment is emerging as an essential parameter in the understanding of the interaction between the tumor and the hosts’ immune system. Since many years the number of TILs has been associated with improved survival and a favourable disease course in various cancers. Galon *et al.* established the “immunoscore”, which is based on the enumeration of CD3+ and CD8+ TILs and was proven to be a strong and independent prognostic tool in CRC [17]. An initial anti-tumoral immune response may be skewed/deviated towards immune tolerance and several mechanisms for this have been reported [18]. It is becoming clear that IDO may play a central role in this, by the induction of anergy in cytotoxic T lymphocytes and stimulation of regulatory T cells [7, 19].

The aim of this research was to study IDO expression in CRC and its relation to disease stage, MMR status and disease relapse. We report a highly consistent IDO expression in the PT, the corresponding TDLN and metastatic tissue of CRC patients, similar to findings in melanoma [8]. Tumoral IDO expression was highly correlated with peri-tumoral expression by host endothelial cells. Of note, this endothelial IDO expression was not limited to tumor-invaded TDLNs, but was also detected in uninvaded TDLNs. The highly consistent expression pattern of IDO, together with the observation that IDO is present in tumor-uninvaded TDLNs, indicates that a climate of immune tolerance may be determined very early in disease.

In melanoma, peri-tumoral IDO expression had a significant impact on the immune cells at the level of the PT and TDLN [8]. In CRC, no significant correlation between IDO expression and CD8+ cells was observed. In a previous study by our group including 265 CRC patients no association was found either [14]. However, normal numbers of CD8 positive cells do not provide information on their functionality. Experiments in rats have demonstrated that CD8+ cells in an IDO-high *in vivo* tissue environment were not eliminated and were still viable, but lost their cytotoxic function [20].

Immunotherapy has dramatically changed the therapeutic landscape in several solid tumors. In CRC, PD-1 and CTLA-4 pathways have been the most studied to date. The potential benefit of PD-1 inhibitors in metastatic patients with a deficient MMR profile was first reported in a phase I trial with nivolumab [21]. Only one patient, who was demonstrated to have a MSI-H tumor, achieved a durable complete response. Improved response rates in MSI-H metastatic tumors were confirmed in a phase II trial with pembrolizumab, with ORR and 20-week-PFS rates of resp. 40% and 78% for MSI-H tumors and resp. 0% and 11% for MSS tumors [22]. These promising results lead to accelerated FDA approval of anti-PD-1 therapy to treat MMR deficient cancer patients who progress on standard therapy.

The potential of PD-L1 as a biomarker for response to PD-1 inhibitors is currently unclear and seems to differ between tumor types. A phase II study demonstrated that responses on nivolumab in CRC patients were independent of the observed PD-L1 expression in MSI-H tumors [23]. These results indicate that PD-L1 is not able to reliably predict clinical benefit of PD-1 blockade in MSI-H CRC patients. Recent phase I studies have been completed with an IDO-inhibitor, epacadostat (INCB024360, Incyte), demonstrating reasonable tolerability and successful inhibition of IDO activity in colorectal carcinoma and melanoma (NCT01195311). Combinatorial data is not yet available for colorectal cancer patients, however encouraging efficacy results of epacadostat with pembrolizumab were obtained in patients with other advanced cancer types (melanoma, renal cell cancer and NSCLC; Keynote 037/NCT02178722). At present it is unknown whether IDO expression can be used as a biomarker for response to IDO inhibition [7].

We report higher IDO expression in tumors with MSI-H compared to MSS. A previous study by Llosa *et al*. demonstrated higher expression of IDO+ TILs in MSI-H tumors. This result was based on RT-qPCR in TILs [24]. Our results support previous findings by Zhang *et al.*, demonstrating elevated tumoral IDO expression in MSI-H CRCs [25]. More important, we report an even more pronounced difference in IDO expression by host endothelial cells in the peritumoral stroma. To the best of our knowledge, higher endothelial IDO expression in MSI-H CRCs has not been reported in literature before. How endothelial IDO expression is induced exactly is currently not fully understood. The increased number of tumor-specific neoantigens in MSI-H tumors, which are induced by frameshift mutations, could be one possible explanation. Next generation sequencing studies have reported that MSI-H tumors harbor 10 to 50 times more mutations than MSS tumors [26]. It is hypothesized that the active immune tumor microenvironment, which is stimulated by this increased neoantigen load, is counterbalanced by the upregulation of IDO, as a negative feedback mechanism with subsequent evasion of these tumors from the immune system. However our data show that IDO expression is not limited to MSI-H CRC alone but can also occur in MSS CRC.

IDO has been reported as an independent prognostic marker in several cancers, including CRC [11–14]. In this study, our data indicate that IDO expression by host endothelial cells is a negative prognostic factor for RFS, independent of disease stage and MMR profile (HR 20.67, 95% CI: 3.05–139.94). Combined presence of endothelial IDO expression and MSS status was associated with the worst prognosis with a median RFS of 4 months.

From a clinical perspective, it is essential to better identify individual patients that will benefit from immunotherapy, in order to reach the best treatment efficacy in individual patients taking into account that these treatments are associated with significant costs and potential toxicities. A recent study in a small group of patients with metastatic renal cell carcinoma demonstrated that endothelial IDO expression was more frequently present in the group of responders to anti-PD1 therapy versus non-responders (100% versus 33.3%) [27]. Our data suggest that IDO-inhibitory strategies might be beneficial in both MSI-H and MSS tumors. These results are supported by a previous study by Angelova *et al*., demonstrating a strong immunological response was counterbalanced by increased gene expression of several immunoinhibitory molecules (CTLA-4, PD-1 and IDO) in both MSI and hypermutated MSS CRCs [28].

Taken together, we demonstrate an early and consistent expression pattern of IDO across different disease stages in CRC patients. The detection of such early signal of immune resistance in cancer could be important as a biomarker for prognosis and (immuno-) therapy response. Although endothelial IDO expression is more frequent in tumors with microsatellite instability, it is associated with decreased RFS, independent of MMR status. These findings indicate that endothelial IDO expression, in addition to MMR status, may be helpful in the stratification of CRC patients in future clinical trials.

## MATERIALS AND METHODS

### Patients

A total of 94 patients who underwent surgical resection of primary colon (*n* = 89) and rectal (*n* = 5) carcinoma and for whom TDLN and metastatic tissue were available were enrolled in this retrospective study. Staging at time of diagnosis was performed according to the AJCC TNM classification (7th edition). Patients who received pre-operative chemotherapy or radiotherapy were excluded from the cohort. Basic patient demographic data are summarized in Table 2. This study was approved by the local ethical committee.

**Table 2:** Patient and tumor characteristics

		Total number of cases	Cohort 1	Cohort 2
**Number of patients, *n***		**94**	**39**	**55**
**Age at diagnosis (median -IQR)**		65 years (55–76)	64 years (54–72)	66 (58–77)
**Gender**				
	Male	55 (58)	24 (62)	31 (56)
	Female	39 (41)	15 (38)	24 (44)
**Clinical stage at diagnosis**				
	Stage I	11 (12)	2 (5)	9 (16)
	Stage II	29 (31)	5 (13)	24 (44)
	Stage III	35 (37)	16 (41)	19 (35)
	Stage IV	19 (20)	16 (41)	3 (6)
**Post-operative chemotherapy** **(No-Folfox-5-FU-Folfiri)**				
	Stage I	11-0-0-0 (100-0-0-0)	2-0-0-0 (100-0-0-0)	9-0-0-0 (100-0-0-0)
	Stage II	19-3-5-2 (66-10-17-7)	3-0-2-0 (60-0-40-0)	16-3-3-2 (67-13-13-8)
	Stage III	12-19-2-2 (34-54-6-6)	4-10-2-0 (25-63-13-0)	8-9-0-2 (42-47-0-11)
	Stage IV	4-11-0-4 (21-58-0-21)	3-10-0-3 (19-63-0-19)	1-1-0-1 (33-33-0-33)
**Tumor location**				
	right-sided	51 (54)	18 (46)	33 (60)
	left-sided	43 (46)	21 (54)	22 (40)
**MMR status^a^**				
	MSS	45 (55)	17 (63)	28 (51)
	MSI-H	37 (45)	10 (37)	27 (49)

Data given are numbers, percentages are given between brackets (%)

^a^The MMR profile could not be determined for 12 cases, due to lack of residual tumor material.

Abbreviations: IQR, interquartile range; MMR status, DNA mismatch repair status; MSI-H, microsatellite unstable; MSS, microsatellite stable.

Formalin-fixed, paraffin embedded (FFPE) primary tumor (PT) tissues of 94 patients were retrieved from the pathology department of the Erasme University Hospital (*n* = 39) and the Ghent University Hospital (*n* = 55). Corresponding tumor draining lymph nodes (TDLNs, *n* = 93) and metastatic tissues (lung or liver, *n* = 27) were retrieved. Both uninvaded and invaded TDLNs were studied (resp. *n* = 43 and *n* = 40). In 10 cases, both were available. Only resections were used for immunohistochemical staining. In order to study differences in IDO expression between MSS and MSI-H CRC and the prognostic significance of IDO expression, only the Ghent University Hospital cohort was used.

MMR status was determined by immunohistochemistry for mismatch repair proteins MLH1, MSH2, MSH6 and PMS2. Normal MMR profile (MSS, *n* = 45) was defined as nuclear expression of all four markers in neoplastic cells and tumors were regarded as MSI-H (*n* = 37) when at least one marker was negative in the presence of a positive internal control.

### Immunohistochemistry

Immunohistochemical staining of 4 μm sections was conducted according to standard avidin-biotin-peroxidase protocols using monoclonal antibodies against IDO (clone 10.1, 1/200, Millipore, Billerica, USA), Foxp3 (clone PCH101, 1/50, eBioscience, San Diego, USA) and CD8 (clone CD8/144B, RTU, Dako). For antigen retrieval, slides were boiled (97° C) for 20 min (PT Link, Dako). A mouse linker (Dako) was added to the protocol in order to amplify the signal of the primary mouse anti-IDO and anti-Foxp3. Antibody detection was visualized using 3-amino-9-ethylcarbazole (AEC) for IDO and diaminobenzidene (DAB) for Foxp3 and CD8 detection. Sections were counterstained with hematoxylin.

### Evaluation of immunohistochemistry

#### IDO

IDO expression was analyzed in neoplastic cells (I) and in endothelial cells in the peri-tumoral stroma (II). (I) In tumoral cells, the intensity of IDO staining was scored semiquantitatively using a four-tiered grading system: no expression (0), weak expression (1+), moderate expression (2+) or strong expression (3+) [14]. For further analysis, tumoral IDO expression was dichotomized into an IDO-low expressing group (0 and 1+) versus an IDO-high expressing group (2+ and 3+). (II) Endothelial IDO expression in the peri-tumoral stroma was scored as “present” or “absent” as earlier described [8].

The scoring procedure was carried out by two independent observers (AM and LV), who were blinded to the patient clinical data. Cohen's kappa value for tumoral IDO expression (4-tiered scale) and endothelial IDO expression were respectively κ = 0.864 and κ = 0.852, which can both interpreted as a ‘near-perfect agreement’ according to Landis and Koch [29].

### FoxP3 and CD8

Stained whole slide tissue sections were digitized and used for automated quantification of Foxp3+ or CD8+ cells. Regions of interest (ROI) were delineated manually per slide. A user-customized algorithm was constructed in Definiens Tissue studio (Definiens Architect XD 64 2.6). Thresholds for cell segmentation, nucleus detection and immunohistochemistry marker intensity were set manually. The fraction of positive cells was calculated as the number of high intensity DAB positive stained cells divided by the total number of cells in the respective tumor areas (=ROIs).

### Statistical analysis

For continuous variables, mean values between 2 groups were compared by the Mann–Whitney *U*-test. The Fisher's exact test was used for the comparison of categorical variables. Cohen's kappa value was calculated to evaluate interobserver agreement for IDO scoring. Recurrence free survival (RFS) was estimated by the Kaplan–Meier method and compared by the log-rank test. RFS was defined as date of diagnosis to date of recurrence or date of death of disease, in case no recurrence was reported previously. Multivariate survival analysis was performed utilizing the Cox proportional hazard model. All statistical analyses were performed using SPSS 24.0 (SPSS Inc, Chicago, IL, USA), a *p*-value (two-sided) less than 0.05 was considered statistically significant.
